# Glances at the Hospitals

**Published:** 1904-09-10

**Authors:** 


### GLANCES AT THE HOSPITALS.

### THE CUMBERLAND INFIRMARY.

The ordinary income amounted to £4,195, and the ordinary expenditure to £5,807, but legacies for £829 were received, and also £1,815 received to pay off the old debt. The average daily number of beds occupied has been 91, which is 10 higher than the average of the previous 10 years; the average duration of the treatment of each in-patient has been 35 days; the average cost per bed occupied was £58 10s., the average cost per patient per day was 3s. 2½d., and the average cost of provisions per patient per annum was £21 2s. The private nursing branch of the hospital yielded a profit of £69. The $x$-ray apparatus has been introduced, shelters for convalescent patients have been put up, and new fireplaces have been put in the wards. At the beginning of the year there were 81 patients in residence and 868 were admitted during it, making a total for the year of 949. Of these 724 were discharged either cured or relieved, 70 for other reasons, 69 died, and 86 remained under treatment. The medical and surgical tables are divided into columns showing the total number of each disease treated, the cured, relieved, and died, and the incurable. A separate table details the operations and gives the results. There were seven amputations of the thigh, all being successful. The operation for appendicitis was performed 23 times, 17 were cured, 4 remained in the hospital, and 2 died. The operation for hernia was performed 14 times, of which 12 were successful, 1 remained under treatment, and 1 died. It will be seen that the tables supply the necessary information upon these points, but we regret to note the absence of an anæsthetic table, and this would have been instructive where 428 operations were performed.

### HASTINGS AND EAST SUSSEX HOSPITAL.

The income for the year amounted to £4,482, and the expenditure was £4,572, leaving a balance against the year's working of about £90; but legacies to the amount of £3,161 were received during the year, so that the finances are in a satisfactory state. At the beginning of the year there were 52 patients in the hospital, and 681 were admitted during the year, making a total of 733 under treatment. Of these, 482 were discharged cured, 50 discharged relieved, 60 for other reasons, and 36 died, leaving 53 in the wards at the end of the year. The medical and surgical statistics are very meagre. The tables merely show the numbers treated for various diseases, and give no results. It is useless to tell us that the operation for appendicitis was performed five times, abdominal section three times, and for perforated gastric ulcer once, unless we are also told what amount of success was obtained. Anæsthetics were administered 290 times, but we are left to guess what agents were used. So much for the medical department, and we do not get much more information from the clerical branch. We failed to find any table showing the cost per head per week or the cost per bed occupied, the average number resident, or the duration of treatment. We earnestly hope that these deficiencies will be seen to in next year's report.

### BIRMINGHAM EAR AND THROAT HOSPITAL.

The total ordinary income amounted to £2,225 7s., and the ordinary expenditure to £2,323 9s., showing a deficit of upwards of £98. The hospital has been considerably added to, and alterations have been made to the older wards whereby they have been made brighter and lighter, and their sanitary condition has been improved. The committee, however, state that the donations to the extension fund amount only to £2,015, and they require a total of £8,000; and the bank account is overdrawn by £2,198. It has therefore been decided to realise some of their investments to partly meet these claims, a necessity which does not speak well for the inhabitants of Birmingham. The total number of beds is now 41; the daily average occupied was 23, and the total number of patients admitted was 781. The tables show no results, and therefore might as well have been left out. We are glad to notice that an anæsthetic table is printed, and from it we learn that chloroform was given 54 times; ether and chloroform, 30; nitrous oxide, 12; ether, 9; ethyl bromide, 3; ethyl chloride, 755; and somnoform twice.

